# Caveolin‐1 associated with severe (pediatric‐onset) presentation of pulmonary arterial hypertension

**DOI:** 10.1002/pul2.12100

**Published:** 2022-07-01

**Authors:** Rachel Farrell, Elizabeth Colglazier, Claire Parker, Leah Stevens, Eric D. Austin, Jeffrey R. Fineman

**Affiliations:** ^1^ Department of Pediatrics UCSF Benioff Children's Hospital San Francisco California USA; ^2^ Department of Pediatrics Vanderbilt University Medical Center Nashville Tennessee USA

**Keywords:** CAV1, pulmonary hypertension, pulmonary vascular disease

## Abstract

There has been a growing interest in the role that genetic factors influence pediatric pulmonary vascular disease. In fact, data suggests that genetic factors contribute to ~42% of pediatric‐onset pulmonary hypertension. Although animal and human studies suggest that aberrations in Caveolin1 (*CAV*1) signaling participate in the development of pulmonary vascular disorders, limited reports of *CAV1*‐associated heritable pulmonary arterial hypertension (HPAH) exist. This is a case report of a 2‐year‐old female with late recognition of HPAH due to a *CAV1* pathogenic variant: c.474del, (p.Leu159Serfs*22)(NM_001753.5). The pedigree demonstrates autosomal dominant transmission with reduced penetrance of PAH, suggestive that additional genetic or environmental factors modify PAH development. Genetic testing and the discovery of rare genetic alterations in PAH during infancy and childhood may aid in identifying disease etiologies, guide therapeutic decisions, and ultimately identify novel therapeutic targets. Moreover, *CAV1* genetics implicate variable expressivity and incomplete penetrance for HPAH and underscores the utility of predictive genetic testing for unaffected family members no matter their age.

## INTRODUCTION

The molecular genetic basis of heritable pulmonary arterial hypertension (HPAH) is evolving and heterogenous, with at least 26 genes currently displaying varying levels of evidence for disease causality. In fact, current data suggests that genetic factors contribute to >40% of pediatric‐onset PAH.^1^ Caveolin 1 (CAV1) is a protein found in the plasma membrane of endothelial cells that is essential for the formation of caveolae. Caveolae are flask‐shaped invaginations of the plasma membrane, whose function modulates many signaling cascades associated with vascular homeostasis, such as the nitric oxide cascade, G‐protein coupled receptors, and the TGF‐β superfamily. Reduced caveolin‐1 expression in lung arterial cells in *CAV‐1*‐related PAH has been reported.^2^, ^3^ Although animal and human studies suggest that aberrations in *CAV1* signaling participate in the development of pulmonary vascular disorders, limited reports of *CAV1*‐associated HPAH exist. This is a case report of a 2‐year‐old female with late recognition of HPAH due to a *CAV1* pathogenic variant: c.474del, (p.Leu159Serfs*22)(NM_001753.5).

## CASE DESCRIPTION

**Figure 1:**
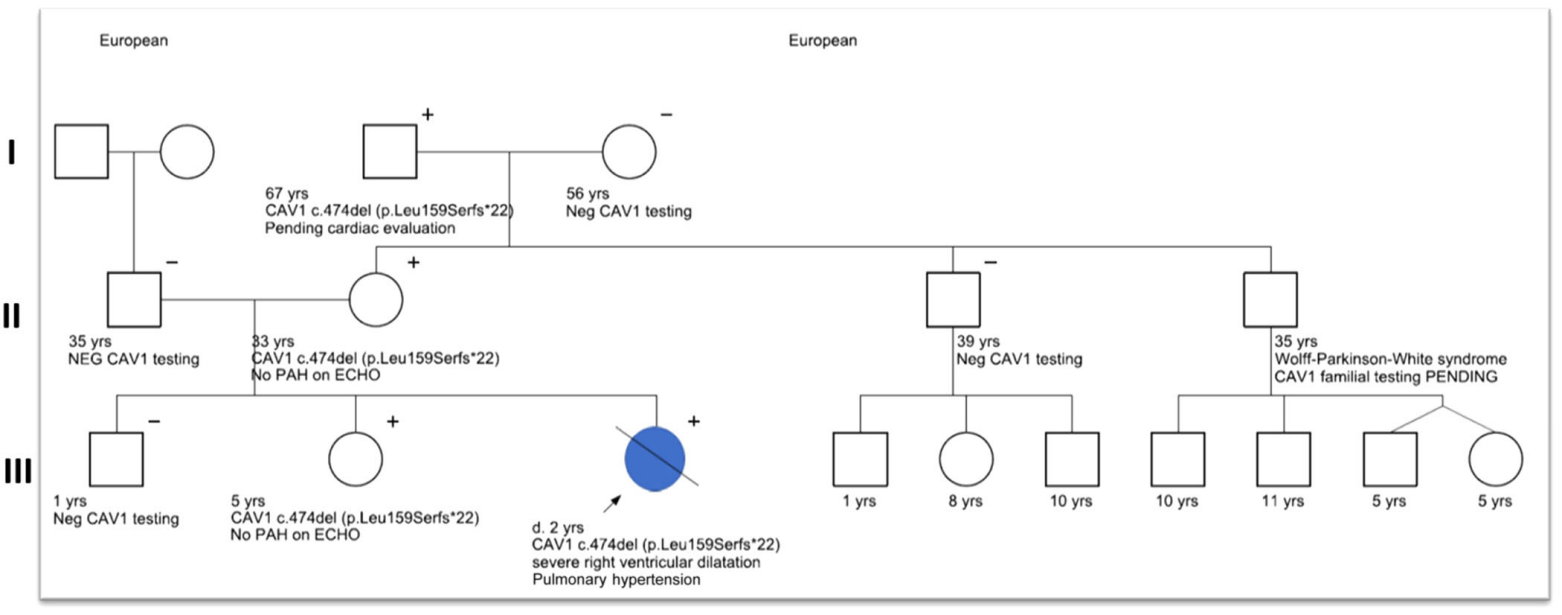
Three‐generation pedigree of family with pulmonary arterial hypertension due to known variant c.474del, p.(Leu159Serfs*22) in CAV1. Eight family members tested for CAV1 variant; four are positive but only one was clinically affected, open square = male; open circle = female; blue circle with slash = affected female deceased. + indicates heterozygous for the c.474delA CAV1 mutation; − indicates negative for CAV1 mutation

Our proband was a 2‐year‐old born full term via NSVD with adequate prenatal care. She had an uneventful birth and neonatal course. During her life, she was evaluated by several pediatric specialists at an outside hospital for reactive airway disease, febrile seizures, thrombocytopenia, and multiple infections including pyelonephritis, conjunctivitis, cellulitis, rotavirus, and acute otitis media. She had no evidence of lipodystrophy. In late December 2019, she had a cardiac arrest at home. She was resuscitated in the field, and stabilized at an outside ED. Evaluation included an echocardiogram, which demonstrated suprasystemic right ventricular (RV) pressure, severely dilated RV, and severe RV dysfunction. She was started on inhaled nitric oxide (iNO) and transferred to UCSF Benioff Children's Hospital in San Francisco. Despite maximal medical treatment with iNO, inhaled iloprost and inotropes, she had marginal hemodynamics (hypotension, tachycardia, CVP 17–25 mmHg). Neurologic evaluation was concerning for extensive hypoxic‐ischemic injury. Given her poor neurological prognosis, all resuscitative efforts were ceased, and she died in her parent's arms. Postmortem genetic testing was sent for Blueprint Genetics Pulmonary Arterial Hypertension panel consisting of sequencing/deletion/duplication analysis of 23 genes. Testing revealed that she carried a rare variant in *CAV1*:c.474del, (p.Leu159Serfs*22). A pedigree of the child's affected family members is presented (Figure 1), notable for additional family members with the variant but no known PAH.

## DISCUSSION

Although animal and human studies suggest that aberrations in *CAV1* signaling participate in the development of pulmonary vascular disorders, limited reports of *CAV1*‐associated HPAH exist. The *CAV1* c.474del, p.(Leu159Serfs*22) variant deletes one base pair in the last exon of *CAV1* and generates a frameshift, leading to a premature stop codon at position 22 in a new reading frame. Of note, as this frameshift variant occurs in the last exon of *CAV1*, the truncated transcript is expected to escape nonsense mediated decay, suggesting that the variant may act as a dominant negative mutation. If so, the persistence of the variant transcript results in a mutated protein product which ultimately promotes pathogenic processes that cause or contribute to the PAH phenotype. There is precedent for this situation in PAH, as we previously reported that mutations which are presumably dominant negative have a more deleterious phenotype in subjects with PAH associated with BMPR2 mutations.^4^ In this circumstance the mutated protein is truncated and predicted to lack the 20 C‐terminal amino acids. This variant is absent from population databases, but has been previously reported by Austin et al., who identified the *CAV1* c.474del, (p.Leu159Serfs*22) variant in 6 individuals within a three generation family who were diagnosed with PAH between the ages of 4 and 67.^5^ An additional three family members carried the variant in that report but were not clinically affected at the time of study, suggesting that penetrance may be reduced for this variant. Western blot analysis from three affected family members showed reduced *CAV1* expression compared to control samples.^5^ Marsboom et al. studied patient‐derived fibroblasts from the family reported by Austin et al.^6^ The variant fibroblasts showed increased proliferation rate associated with hyperphosphorylation of SMAD 1/5/8 and inhibition of the antiproliferative function of CAV1. Expression of the truncated CAV1 protein in null mouse fibroblasts showed the variant protein failed to induce formation of caveolae due to retention in the endoplasmic reticulum, again suggesting variable expressivity and incomplete penetrance for PAH. In separate work using this and other *CAV1* variations experimentally, Gairhe et al. demonstrated the important role that *CAV1* signaling plays in protecting human pulmonary arterial endothelial cells from many of the pathologic hallmarks of PAH, including exuberant proliferation, impaired apoptosis, dysregulated cellular migration, and perturbed inflammatory response to insult.^7^

In our particular case, three generations of family members have been tested for the *CAV1* variant, and of the eight family members tested, only one of four individuals testing positive for the variant expressing the PAH phenotype. Her disease was acute and severe and resulted in her untimely death at 2 years of age after a series of inflammatory insults associated with infection. The correct identification of her *CAV1* variant has allowed identification of those who are genotype positive in the family to seek appropriate cardiac surveillance to monitor for signs of PAH. In general, the penetrance of the majority of disease‐causing PAH genes is not well known. For example, penetrance for *BMPR2* variants has been estimated at ~20% overall with sex‐dependent penetrance due to higher penetrance observed in female (42%) versus male (14%) carriers.^8^, ^9^ However, penetrance for individuals with hereditary PAH due to variants in *ACVRL1, KCNK3, CAV1, SMAD9,* or*BMPR1B* is not yet known. Pathogenic variants are identified in about 20%‐30% of patients with idiopathic PAH, and in about 75% of patients with familial PAH, although these percentages are likely higher in those diagnosed during childhood.^1^, ^10^, ^11^, ^12^ Genetic testing can be useful for determining familial risk assessment as well as inheritance if a pathogenic variant is identified. *De novo* genetic variants contribute to a significant proportion of pediatric PAH cases, approximately 15%, suggesting the presence of other genetic, epigenetic and environmental factors interact with one's risk for PAH.^1^ It remains unclear whether inflammatory insults are one such trigger for *CAV1* variant carriers. Given the incomplete penetrance of PAH genes, including *CAV1*, predictive testing can illuminate who may be truly at risk. However, given the incomplete clinical sensitivity for genetic testing for familial PAH, a negative result does not rule out a genetic cause or contribution to a patient's PAH.

This case report demonstrates HPAH associated with *CAV1* variant in childhood presents with severe disease and poor survival, as a prior study reported.^5^ The pedigree demonstrates autosomal dominant transmission with reduced penetrance of PAH, suggestive that additional genetic or environmental factors modify PAH development as seen in other heritable forms of PAH (e.g., BMPR2 gene mutations). In this case, neither comprehensive exonic nor genomic sequencing were performed to evaluate for additional variants in the genome which may have contributed to the expression of PAH. However, it is possible, and perhaps likely, that such genomic variations do exist. In fact years of advances in the field at the DNA, RNA, and protein levels, including a recent multinational study focused on outcomes among those with PAH, suggest this is true.^13^ Synergistic interactions between the CAV1 mutation, additional molecular variations, and other influences such as intermittent inflammatory insults may have contributed to the severity of her course.^14^ Related to this, active research studies incorporating whole genome and/or whole exome sequencing of all members of families impacted by mutations in genes associated with PAH are ongoing in our laboratories and others to determine the potential genetic factors which modify disease penetrance, severity, and outcomes. Genetic testing and the discovery of rare genetic alterations in PAH during infancy and childhood may aid in identifying disease etiologies, guide therapeutic decisions and improve outcomes, identify family members at risk, and ultimately identify novel therapeutic targets. Moreover, *CAV1* genetics implicate variable expressivity and incomplete penetrance for HPAH and underscores the utility of predictive genetic testing for unaffected family members no matter their age.

## AUTHOR CONTRIBUTIONS

All authors have made substantial contributions to the conception of the work; revising the work critically for important intellectual content; have approved the final version to be published; and agree to be accountable for all aspects of the work in ensuring that questions related to the accuracy or integrity of any part of the work are appropriately investigated and resolved.

## CONFLICT OF INTEREST

The author declares no conflict of interest.

## ETHICAL STATEMENT

This study was performed under an Institutional IRB approval.
